# Very young infants learn abstract rules in the visual modality

**DOI:** 10.1371/journal.pone.0190185

**Published:** 2018-01-02

**Authors:** Brock Ferguson, Steven L. Franconeri, Sandra R. Waxman

**Affiliations:** Department of Psychology, Northwestern University, Evanston, IL, United States of America; Universidad de Salamanca, SPAIN

## Abstract

Abstracting the structure or ‘rules’ underlying observed patterns is central to mature cognition, yet research with infants suggests this far-reaching capacity is initially restricted to certain stimuli. Infants successfully abstract rules from auditory sequences (e.g., language), but fail when the same rules are presented as visual sequences (e.g., shapes). We propose that this apparent gap between rule learning in the auditory and visual modalities reflects the distinct requirements of the perceptual systems that interface with cognition: The auditory system efficiently extracts patterns from sequences structured in time, but the visual system best extracts patterns from sequences structured in space. Here, we provide the first evidence for this proposal with adults in an abstract rule learning task. We then reveal strong developmental continuity: infants as young as 3 months of age also successfully learn abstract rules in the visual modality when sequences are structured in space. This provides the earliest evidence to date of abstract rule learning in any modality.

## Introduction

Humans are adept pattern learners, tracking the co-occurrences and frequencies of input in myriad domains [1,2]. But our most striking cognitive advances in language, science, and mathematics rest on our ability to detect the higher-level ‘abstract rules’ underlying these observed patterns [3–5]. A hallmark of higher-level rules is that they are not tied to the particular sequences of perceptual input from which they were gleaned but instead, are sufficiently abstract to be generalized to entirely novel elements [6]. Infants draw on such rules early in development, as witnessed by their success in acquiring the rules of language [7], rules governing the behavior of physical objects [8], and rules guiding social interactions [9].

Nonetheless, there is considerable debate concerning the origins of abstract rule learning in infancy, and the breadth of stimuli that the learning mechanism takes as input. Central to this debate is the observation that human infants succeed in learning rules from some kinds of input but not others. From birth, infants reliably detect abstract rules in auditory input, including speech and tone sounds [10–13]; however, it is not until 7 months of age that infants are able to detect the same rules from visual input [14–18]. This pattern—protracted rule learning from visual input relative to auditory input—has been interpreted as evidence that initially, abstract rule learning is possible only over a restricted range of stimuli [14,17,19].

Here we propose a different view: that this apparent gap between auditory and visual rule learning reveals less about limitations on infants’ capacity to learn rules *per se* than it does about the specific requirements of the perceptual systems which interface between cognitive processes and the stimuli over which they operate. More specifically, we propose that these differences between infants’ rule learning in the auditory versus visual modalities reflect important differences in the ways that the auditory and visual perceptual systems best extract patterns from input.

Although the auditory system most effectively extracts statistical patterns from sequences that are structured in *time* (i.e., in which one element follows another, each occurring one-at-a-time), the visual system best extracts patterns from sequences that are structured in *space* (i.e., in which the sequence of elements appear in different locations and, even briefly, allow for simultaneous observation of all elements) [20–23]. Although statistical patterns differ in important respects to abstract rules [6], it is nonetheless important to note that most studies of rule learning in the visual modality have presented visual sequences with temporal, but not spatial, structure. And, in all such cases, infants reveal stark failures or inconsistent learning throughout their first year [15,16,18]. In only two investigations were infants permitted to observe the elements in the sequence simultaneously; in both, infants successfully detected rules [14,17].

This pattern of successes and failures is consistent with our hypothesis, but does not on its own provide sufficiently strong evidence. First, there is an experimental confound. Studies in which infants have succeeded versus failed in rule learning have not only differed in presentation structure but also in the elements comprising the sequences. Each experiment documenting a failure constructed sequences using geometric shapes; each experiment documenting a success used animate objects (dogs, cats, or humans). This leaves open the possibility that it is not the format in which sequences are presented that matters, but rather characteristics of the individual elements themselves [14,17].

Second, there is a developmental gap. By 4 months, infants successfully learn abstract rules in the auditory modality [12], but it is not until 7 months that we see evidence for successful abstract rule learning in the visual modality [14]. This presents a puzzle: If 4-month-old infants can learn rules in the visual modality when the sequences are presented with spatial structure, then why would they not–under this more ideal presentation condition–successfully abstract rules in the visual modality as well?

Here we test our proposal that (1) the visual system most effectively extracts patterns from spatially-structured, simultaneously-observed input, and that (2) this is a fundamental capacity of the visual system that is continuous throughout development. In Experiments 1 and 2, we tested adults’ and 3- and 4-month-olds infants’ rule learning using images of dogs as sequence elements. In Experiments 3 and 4, we used the same experimental design to examine their performance with geometric shapes as sequence elements. These experiments permitted us to uncover the independent influences of the structure in which sequences are presented, and the elements comprising the sequences.

## Experiment 1

Although we know that the visual system extracts statistical patterns from visual stimuli most effectively from spatially-structured input, there is no evidence of an advantage for spatially-structured input in the domain of abstract rule learning. Thus, before turning to infants, we first designed a new task to compare adults’ detection and generalization of abstract rules from visual sequences in three different presentation modes: temporal structure alone (T), temporal plus spatial structure (TS), and temporal plus spatial structure combined with an opportunity for simultaneous comparison of the sequence elements (TSS). If the visual system’s preference for spatially-structured, simultaneously-observable input extends to abstract rules, then adults should most effectively detect and generalize rules in the TSS condition.

### Method

#### Participants

Thirty-six adults recruited via Amazon’s Mechanical Turk participated. Another 5 adults were excluded for low accuracy (less than 70% overall) and one was excluded for failing to contribute data in at least 40 trials in each condition after trials with excessively long response times (greater than 2.5 seconds) were excluded. Our sample size was selected on the basis of a pilot study (*N* = 17) indicating an average accuracy effect size between conditions of approximately *d* = 1.00. With *N* = 36, we estimated 99.99% power to detect a difference. Our low-accuracy exclusion criterion was also established during this pilot, in which we observed a steep accuracy drop-off below 70%, suggesting that participants below this criterion were not attending to the task.

#### Materials

To permit comparison with prior work [14], twelve images of unique dog breeds were used to construct three-element sequences. Eight of the dogs (an *Alaskan Malamute*, *Norweigan Elkhound*, *Shiba Inu*, *Nova Scotia Duck Tolling Retriever*, *Australian Cattle Dog*, *Belgian Malinois*, *Canaan*, and *German Shepherd*) were used to construct initial sequences; the four remaining dogs (a *Finnish Spatz*, *Akita*, *Anatolian Shepherd*, and *Belgian Tervuren*) were used to construct test sequences.

#### Procedure

Each participant completed 144 trials of a “pattern detection game” in their web browser. On each trial, the participant was shown an initial sequence of images arranged to form either an AAA, AAB, ABB, or ABA pattern. Our crucial manipulation was the way in which this sequence was revealed: In a *temporal* (T) condition, each image appeared in sequence for 150 msec in the same location at the centre of the screen. In a *temporal+spatial* (TS) condition, each image appeared in sequence for 150 msec in three distinct side-by-side locations. In a *temporal+spatial+simultaneous* (TSS) condition, each image appeared in the same three distinct side-by-side locations as in the TS condition; however, in the TSS condition, the images remained on the screen such that all three were availably briefly (for 150 msec) for simultaneous observation. In all three conditions, each image appeared 175 msec after the previous image appeared (leaving a 25 msec blank screen between images in the T and TS conditions).

After the final image had been shown for 150 msec, participants saw a white screen for 500 msec before a test sequence comprised of new images appeared. All images were presented simultaneously. Participants then indicated whether the test sequence followed the “same” or “different” pattern as the initial sequence using the ‘S’ and ‘D’ keys on their keyboards. They were not given feedback. After 1000 msec, the next trial began.

Participants completed 48 trials in each condition and, within each condition, there was a 50% chance that the test sequence matched the initial sequence. Trial order was randomized within-subjects, as were the images used to the construct the sequences. Prior to beginning the task, participants were instructed on how to play the game and completed 10 practice trials to ensure their understanding.

#### Predictions

We expected that, if adults most effectively extract and generalize rule-based patterns (as they do statistical patterns) from spatially-structured, simultaneously-observable input they would be most accurate and faster to respond in TSS trials than both T and TS trials.

All stimuli and methods for each of the experiments described in this paper were approved by the Institutional Review Board at Northwestern University.

### Results

#### Accuracy

Overall, participants were 89% correct in indicating whether test sequences followed the same or a different pattern than the initial sequence. A repeated-measures ANOVA assessing the effect of condition on subjects’ accuracy (proportion correct) yielded reliable differences between conditions (*F*(2,70) = 29.02, *p* < .001). Post-hoc pairwise t-tests (dependent samples) indicated that as predicted, participants were more accurate in TSS trials (*M* = 95.52%) than in either TS trials (*M* = 85.87%, *M*Δ = 9.65% [95% CI: 7.19, 12.11], *t*(35) = 7.98, *p* < .001, *d* = 1.33) or T trials (*M* = 86.34%, *M*Δ = 9.20% [95% CI: 6.25, 12.13], *t*(35) = 6.34, *p* < .001, *d* = 1.06). Accuracy in TS and T trials did not significantly differ (*M*Δ = .46% [95% CI: -2.79, 3.71], *t*(35) = .29, *p* = .77, *d* = -.048).

#### Response times

Participants’ response times provided additional support for the TSS advantage. They responded more quickly in TSS trials (*M* = 828 msec) than in TS trials (*M* = 876 msec, *M*Δ = 48.16 [95% CI: 17.32, 79.01], *t*(35) = 3.17, *p* = .0032, *d* = -.53) or T trials (*M* = 880 msec, *M*Δ = 52.46 [95% CI: 23.06, 81.87], *t*(35) = 3.62, *p* < .001, *d =* -.60). Response times in TS and T trials did not significantly differ from each other (*M*Δ = 4.30 [95% CI: -21.92, 30.52], *t*(35) = .33, *p* = .74, *d* = -.056).

### Discussion

These results provide the first evidence that the visual system most effectively detects and generalizes abstract rules when the sequences are temporally- and spatially-structured, providing an opportunity for simultaneous observation.

It is possible that the advantage we report for simultaneously-observed sequences is due to this element of our design; that is, participants did better after observing the training sequence simultaneously because they were tested on a simultaneously-presented sequence. We therefore ran a control experiment (*N* = 34) in which participants were tested on T sequences. If our results were due to a match between training and test format, we should have seen a T advantage in this experiment. Instead, we saw precisely the same accuracy pattern: an overall main effect of Condition (*F*(2,66) = 6.51, *p* = .0026), with greater accuracy in TSS than either TS (*p* = .002) or T (*p* = .0033), and no difference between TS and T (*p* = .69).

These findings lay a foundation for asking whether very young infants can successfully detect and generalize abstract rules in the visual modality under these conditions.

## Experiment 2

Here we addressed the developmental gap between rule learning in the auditory and visual modalities. We know that 4-month-olds can learn rules in the auditory modality [12], but can they also do so in the visual modality when sequences are spatially-structured and simultaneously observable? To address this question, we assessed 3- and 4-month-olds’ rule learning using a habituation visual rule learning paradigm first introduced by Saffran and colleagues (2007).

### Method

#### Participants

Subjects were 40 3- and 4-month-old infants (23 males, ranging 3.00–4.97 months). An additional 26 infants were excluded and replaced due to fussing out prior to reaching the test phase (*N* = 14), failing to complete at least two familiar and two novel trials (*N* = 7), technical error (*N* = 3), irritability during test (*N* = 1), or parental interference (*N* = 1). Although we had initially planned to run 20 infants (based on the sample sizes of comparable findings; [6,14], upon analysis of these data, we opted to double our sample size to permit us to pursue an intriguing hint of a developmental transition between 3 and 4 months. Because not following *a priori* stopping criteria can inflate the likelihood of making a Type I error, we address these concerns here by noting that infants’ overall familiarity preference (our critical measure), was statistically significant in the first sample alone, (*M* = -2.04 sec, *SD* = 4.13, *t*(19) = -2.20, *p* = .04).

#### Visual stimuli

Identical to Experiment 1 and Saffran et al. (2007). In this case, the dog images used in the initial sequences in Experiment 1 were used during the habituation phase, organized into *A* and *B* categories. The A elements were the *Alaskan Malamute*, *Norwegian Elkhound*, *Shiba Inu*, and *Nova Scotia Duck Tolling Retriever*, and the B elements were the *Australian Cattle Dog*, *Belgian Malinois*, *Canaan Dog*, and *German Shepherd*. The dog images used in the test sequences in Experiment 1 were used during the test phase. Here, the A elements were the *Finnish Spatz* and *Akita*, and the B elements were the *Anatolian Shepherd* and *Belgian Tervuren*.

#### Procedure

Infants participated in both a habituation and test phase. During habituation, infants saw sequences of dog images on a screen that each followed either an ABB or ABA rule, randomized between-subjects. Each dog appeared in sequence in 330 msec intervals and remained visible thereafter. After the third dog in each sequence appeared, all three dogs remained on the screen for 1840 msec. During this time, half of the infants observed the sequence in silence while the other half heard a phrase of infant-directed speech (either “Look at the *toma*!” or “Do you see the *toma*?” randomized across trials). Prior work has revealed that listening to speech facilitates infant learning [24], thus we introduced this manipulation here to explore whether listening to speech augments rule learning. A blank screen separated each sequence by 500 msec. For a given trial, triads were displayed until infants looked away from the screen for 2 sec. This signaled the end of a trial; at this point, an attention-getter appeared at the screen center. When infants looked to the attention-getter for 250 msec, a new habituation trial began. The habituation phase continued until infants habituated, which occurred either when (1) their average looking time across three consecutive trials fell below 50% of their looking time during the first three trials, or (2) when infants had viewed the maximum number (25) of habituation trials (only two infants hit the maximum).

Next, infants participated in a test phase consisting of 8 trials, all presented in silence. In each, infants viewed sequences comprised of novel dog images. In half of the trials, the dogs were organized in an ABB pattern while, in the other half of trials, the dogs were organized in a ABA pattern, thus resulting in trials that matched either the familiar (habituated) rule or a novel rule. Trials were presented in two blocks of four; within each block, the order of familiar rule and novel rule trials was randomized. Test trials were identical in timing as the habituation trials.

#### Coding and data preparation

Using custom MATLAB software, each infant’s looking time to the screen was coded online by a trained observer blind to our hypotheses. Prior to analysis, test trials were excluded in which infants looked less than 2.5 seconds to the screen (the length of a single sequence) so that they could discriminate novel from familiar sequences (excluding 57 trials, or 17% of all trials).

#### Predictions

If infants successfully learned the rule presented during habituation (e.g., AAB), then at test, they should distinguish novel examples of the familiar rule (AAB) from examples of novel rules (ABA) and should reveal a preference for one over the other. The typical prediction in habituation designs is that, if infants successfully learn during the habituation phase, they will prefer novel the novel stimuli at test. However, we did not have *a priori* expectations about the direction that infants’ preferences might take for three reasons. First, the current experiment is the first to present such young infants with these particular visual sequences (images of dogs). Second, unlike traditional habituation experiments in which the ‘familiar’ test stimuli are identical to those to which infants were habituated, our design presented novel images in both familiar and novel test trials (only the abstract rule was familiar to habituated infants). Third, infants in rule learning studies more generally have found variability in whether infants express their learning as a familiarity or novelty preference across various stimuli and designs (e.g., Saffran, 2007; Thiessen, 2012). Infants’ directions of test preferences in designs like these may in fact provide insight into the processing load inherent in the task. Decades of research reveal that infants are more likely to favor the familiar test stimulus when they are very young or when stimuli are relatively complex and difficult to process, but infants tend to favor the novel test stimulus when they are older or when stimuli are simpler to process [25]. Evidence for this shift from familiarity to novelty preferences has been found in several domains, including object categorization [24] and abstract rule learning [11,26]. In the present design, then, a preference for the familiar rule at test would suggest that, although infants successfully learned the rule during habituation, this task imposed a significant load on their nascent cognitive abilities.

### Results

#### Habituation phase

Infants habituated in 9.68 trials (*SD* = 5.00) on average. Neither the number of trials to habituate (*t*(38) = 1.11, *p* = .27) nor total seconds to habituate (*M* = 126.34, *SD* = 68.88, *t*(38) = .98, *p* = .33) varied as a function of auditory context (i.e., whether habituation trials were presented with speech or in silence).

#### Test phase

**Fig 1:**
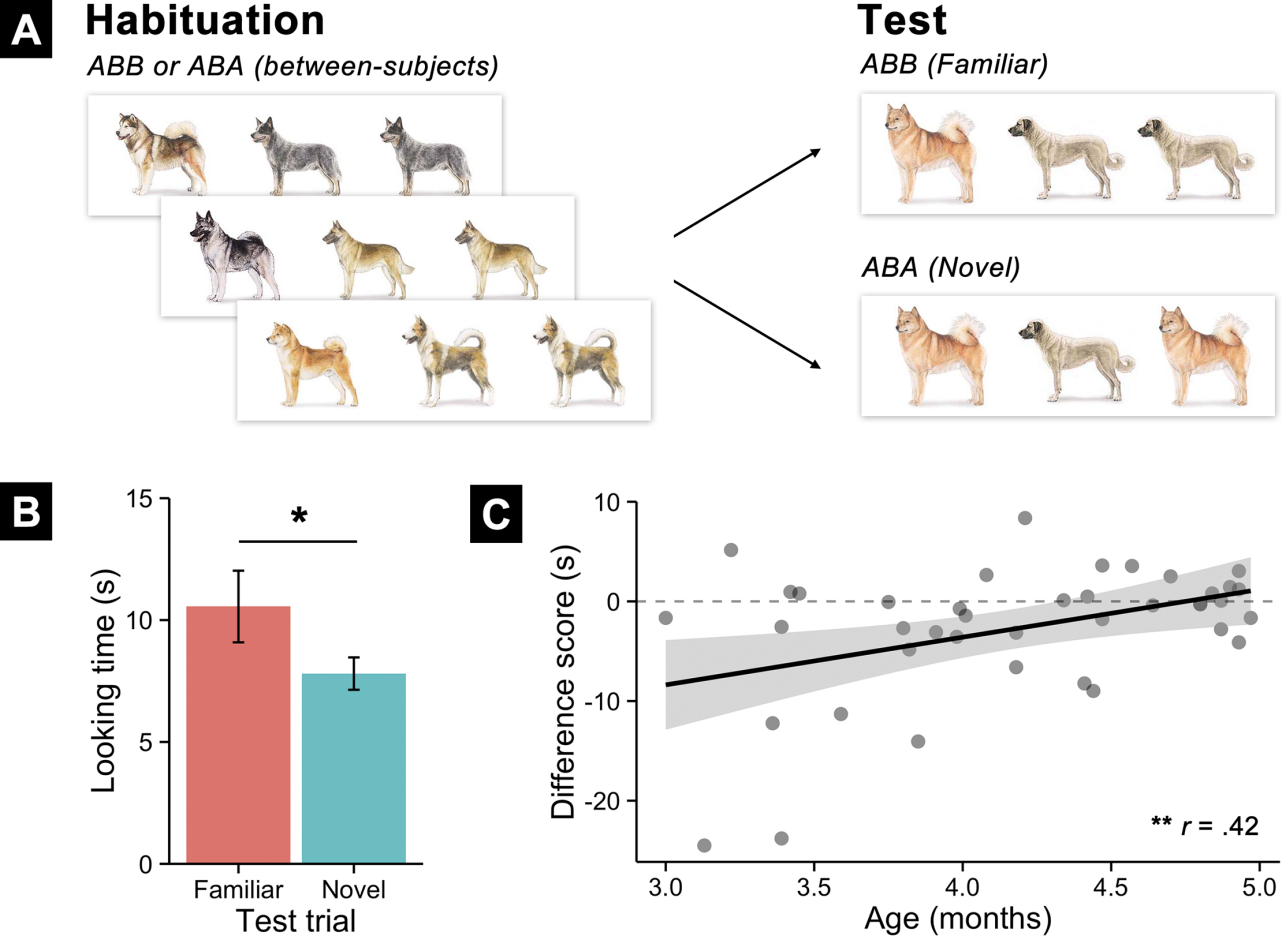
**Experimental design (A) and results (B-C) for Experiment 2 with 3- and 4-month-old infants.** (A) Infants were habituated to sequences of dogs following a rule (either ABB or ABA). After habituation, to test whether they learned the rule, they were shown two types of new sequences: familiar sequences that followed the habituated rule and novel sequences that followed a different rule. (B) Infants’ looking preferences at test by trial type, revealing a significant preference for familiar rule trials, documenting that they learned the rule during habituation. (C) Infants’ difference scores (novel–familiar looking) plotted by age. Older infants were less likely to show a familiarity preference then younger infants, suggesting that rule learning from dogs became easier with age.

Infants successfully detected the rule during habituation and generalized it to novel sequences at test (see Fig 1). A 2 (Test Type: Familiar vs. Novel) x 2 (Auditory Context: Speech vs. Silence) repeated-measures ANOVA predicting infants’ looking times at test revealed a significant main effect of Test Type (*F*(1,38) = 6.49, *p* = .015) but no main effect or interaction with Auditory Context (both *F*’s < 1). Infants looked longer to test sequences following the familiar rule (*M* = 10.56 s, *SD* = 9.29) over the novel rule (*M* = 7.80 s, *SD* = 4.20), *M*Δ = -2.75 [95% CI: -4.92, -.59], *t*(39) = -2.57, *p* = .014, *d =* -.41. However, the strength of infants’ familiarity preferences declined with age (*r* = .42, *p* = .0075). This developmental pattern converges with evidence that when young infants are engaged in tasks that are particularly challenging, they tend to favor familiar over novel test stimuli, but that with age, as their processing efficiency increases and the same tasks become less challenging, infants tend to favor novel over familiar test stimuli (e.g., [24].

Several additional analyses provided converging evidence for this interpretation: First, a Wilcoxon (non-parametric) signed rank test revealed a significant difference between novel and familiar trials, V = 572, *p* = .029. Second, we asked whether infants dishabituated (i.e., looked longer) to either novel or familiar trials than their average looking during the last 3 trials of habituation. This would indicate that when presented with the test stimuli, they noticed a difference that reignited their interest. Infants dishabituated to familiar test trials (*t*(39) = 2.52, *p* = .015) but not to novel trials (*t*(39) = 1.43, *p* = .16). Finally, we used a series of linear mixed-effects models to analyze infants’ looking trial-by-trial. These allowed us to estimate the effects of Test Type, Auditory Context, and Age while controlling for trial order and random differences between participants in their baseline attention (i.e., allowing their intercepts to vary) and responsiveness to fixed factors (i.e., allowing slopes for Test Type and Trial Order to vary). Model comparisons revealed reliable main effects of Test Type (*β* = -2.80, *SE* = 1.32, *χ*^2^(1) = 4.47, *p* = .034), Age (*β* = -5.76, *SE* = 1.52, *χ*^2^(1) = 12.88, *p* < .001), and a Test Type by Age interaction (*β* = 5.00, *SE* = 2.30, *χ*^2^(1) = 4.74, *p* = .029). Overall, infants preferred familiar test stimuli, but the strength of this preference decreased with age, as did infants’ overall looking time.

### Discussion

When visual sequences are structured in space, and are available even briefly for simultaneous observation, infants as young as 3 months successfully abstract rules and generalize them to novel sequences of new images at test. Nevertheless, an overall preference for the familiar test sequences indicates that this task carried a substantial processing burden for infants so young. What is the source of this burden? One possibility is that rule learning in the visual domain is itself difficult for very young infants. Another possibility is that the complexity of the visual elements (images of dogs), rather than rule learning in the visual domain per se, introduced the processing burden. If this is the case, then instantiating the same rules with visual elements that are less complex should relieve infants and permit them to reveal novelty preferences at test.

## Experiment 3

Our goal was to identify a set of elements that were easier to process than images of dogs. Although we imagined that images of geometric shapes would satisfy this criterion, others have argued that infants learn abstract rules more easily from images of dogs than from shapes because dogs are more familiar [14,17]. Therefore, before testing infants with geometric shapes, we first examined adults’ performance in the same task as Experiment 1, replacing the images of dogs with shapes. If processing sequences of shapes is less burdensome than sequences of dogs, then adults should respond with greater speed and accuracy here than in Experiment 1.

### Method

#### Participants

A new group of 36 adults recruited via Amazon’s Mechanical Turk completed our task. An additional 7 participants were excluded for inattentiveness indicated either by low accuracy (below the 70% accuracy criterion established during our pilot) and two were excluded for failing to contribute at least 40 trials in each condition after trials with excessively long response times (greater than 2.5 seconds) were excluded.

#### Materials

Twelve solidly-coloured shapes were used to construct the three-element sequences. Eight of the shapes (an *octagon*, *chevron*, *bowtie*, *triangle*, *square*, *diamond*, *4-pointed star*, and *circle*) were used to construct initial sequences; four of the shapes (a *5-pointed star*, *clover*, *cross*, and *crescent*) were used to construct test sequences.

#### Procedure

Identical to Experiment 1.

#### Predictions

First, we expected to replicate our overall effect from Experiment 1: participants should be most accurate in generalizing rules when the initial sequence was presented in TSS format. Second, if detecting rules from shapes is easier than from dogs, then we expected to see a main effect of Experiment on accuracy: participants should be more accurate here than in Experiment 1.

### Results

#### Accuracy

Overall, participants were 94% accurate in indicating whether the test sequence followed the same or different pattern as the initial sequence. Nevertheless, a repeated-measures ANOVA determining whether subjects’ accuracy (proportion correct) varied by condition yielded reliably differences between conditions (*F*(2,70) = 12.01, *p* < .001). Post-hoc pairwise t-tests (dependent samples) revealed the same pattern of results as in Experiment 1: Participants were more accurate in TSS trials (*M* = 96.18%) than both TS (*M* = 93.60%, *M*Δ = 2.59% [95% CI: .09, 4.27], *t*(35) = 3.12, *p* = .0036, *d* = .52) and T (*M* = 91.93%, *M*Δ = 4.25% [95% CI: 2.43, 6.07], *t*(35) = 4.73, *p* < .001, *d* = .79) trials. TS and T trials did not significantly differ (*M*Δ = 1.67% [95% CI: -.15, 3.48], *t*(35) = 1.86, *p* = .071, *d* = .31). Critically, a hierarchical linear model predicting participants’ accuracy scores by Condition and Experiment (1 vs. 3) indicated a main effect of Experiment (*β* = .47, *SE* = .15, *χ*^2^(1) = 9.91, *p* = .0016): As predicted, participants were more accurate when detecting rules from shapes (*M* = 93.90%) than dogs (*M* = 89.24%).

#### Response times

As in Experiment 1, participants’ response times indicated an advantage for TSS trials. Participants were faster to respond in TSS trials (*M* = 823 msec) than in T trials (*M* = 887 msec, *t*(35) = 5.12, *p* < .001, *d =* -.85) or TS trials (*M* = 829 msec); this latter comparison was not significant (*t*(35) = .55, *p* = .58). TS trials were also faster than T trials (*t*(35) = 5.60, *p* < .001, *d* = -.93). A hierarchical linear model indicated that, despite the significant increase in accuracy in Experiment 3 over Experiment 1, participants were equally fast to respond in the two Experiments (*β* = -15.63, *SE* = 40.64, *χ*^2^(1) = .15, *p* = .70). Thus, although they were no faster here than in Experiment 1, this result provides reassurances that participants’ increased accuracy with shapes (over dogs) did not come at the expense of decreased speed (i.e., a speed-accuracy tradeoff).

### Discussion

Adults more accurately detected and generalized rules from sequences of geometric shapes than dogs. This suggests that the images of shapes imposed a lower processing burden than did the perceptually more complex images of dogs. In the next experiment, we ask whether the same is true for infants.

## Experiment 4

We presented a new group of 3- and 4-month-old infants with sequences constructed from images of geometric shapes (identical to Experiment 3) matched to the studies in which 5-, 8-, and 11-month-olds have failed to learn rules in earlier studies [15,16]. By considering these results in conjunction with those of Experiment 2, we can assess the influence of the individual elements as well as their structure on visual rule learning. If spatial structure is critical, then infants should successfully extract abstract rules here because, unlike prior studies, we present the geometric shapes in an optimal format. Moreover, if shapes impose less of a burden on infants’ rule learning than dogs, then infants here should prefer the novel test sequences.

### Method

#### Participants

Subjects were 20 3- and 4-month-old infants (6 males, ranging 3.16–4.77 months). An additional 8 infants were excluded and replaced due to fussing out prior to reaching the test phase (*N* = 6), or exhibiting a preference (calculated as a difference score) at test 5+ *MAD* and 2.5+ *SD* from the overall mean (*N* = 2).

#### Visual stimuli

Shape images were organized into *A* and *B* categories. During habituation, the A elements were an *octagon*, *chevron*, *bowtie*, and *triangle*, and the B elements were a *square*, *diamond*, *four-pointed star*, and *circle*. At test, the A elements were a *five-pointed star* and *clover*; the B elements were a *cross* and *crescent*.

#### Procedure

Identical to Experiment 1.

#### Coding and data preparation

Each infant’s looking time to the screen was once again coded online by a trained observer blind to our hypotheses. Removal of test trials less than 2.5 seconds (the length of one sequence) resulted in the loss of 20 trials (12.5%).

#### Predictions

If infants successfully detected the rule during habituation and generalize this rule to discriminate the test sequences, we expected that they would look longer to one type of test trial over the other. If learning rules from shapes imposes the same processing burden as from dogs, then we predicted that infants would once again prefer familiar trials at test. In contrast, if learning rules from shapes imposes less of a processing burden than dogs, we predicted that their preference would reverse; that is, they would show a preference for novel trials at test.

### Results

#### Habituation phase

Infants habituated in 10.60 trials (*SD* = 6.83) on average. As in Experiment 2, neither total habituation trials (*t*(18) = 1.26, *p* = .23) nor total seconds to habituate (*M* = 160.87, *SD* = 108.50, *t*(18) = -1.73, *p* = .10) varied by auditory context. Moreover, neither of these habituation metrics differed between Experiments 2 and 4 (both *p*’s > .20), suggesting that infants’ attention during habituation was equivalent, whether they were viewing images of dogs or shapes.

#### Test phase

**Fig 2:**
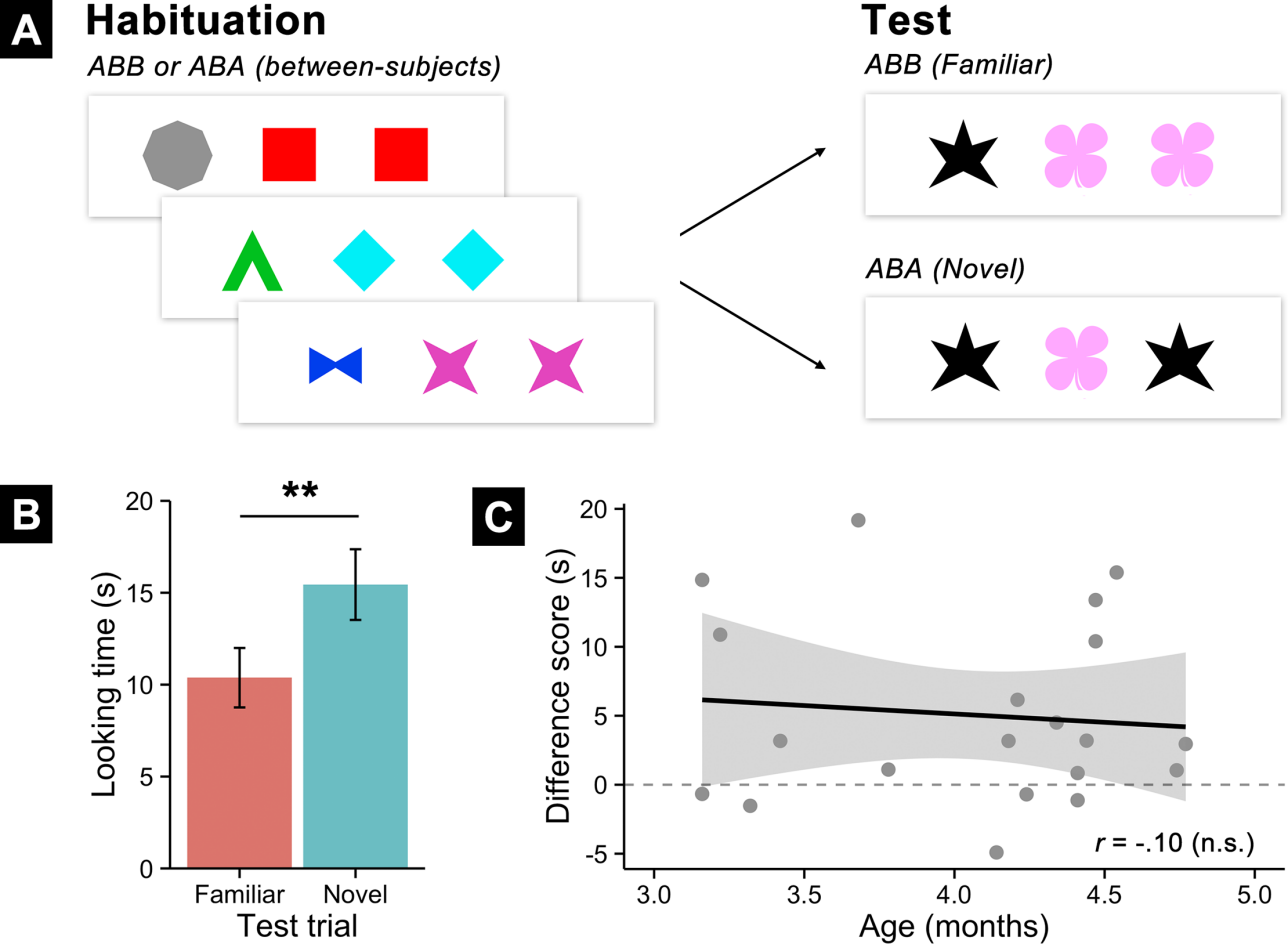
**Experimental design (A) and results (B-C) for Experiment 4 with 3- and 4-month-old infants.** (A) Infants were habituated to sequences of colored shapes following a rule (either ABB or ABA). After habituation, to test whether they learned the rule, they were shown two types of new sequences: familiar sequences that followed the habituated rule and novel sequences that followed a different rule. (B) Infants’ looking preferences at test by trial type, revealing a significant preference for novel rule trials, once again documenting that they learned the rule during habituation. (C) Infants’ difference scores (novel–familiar looking) plotted by age. Infants at all ages were equally likely to show a novelty preference, suggesting that rule learning from shapes did not become easier during this timeframe.

Infants successfully learned abstract rules from sequences of shapes (see Fig 2). A 2 (Test Type) x 2 (Auditory Context) repeated-measures ANOVA predicting infants’ looking times revealed a significant main effect of Test Type (*F*(1,18) = 11.14, *p* = .0037), but no main effect or interaction with Auditory Context (both *F*’s < 1). Follow-up tests confirmed that, as predicted, infants in this experiment revealed a preference for novel (*M* = 15.44 s, *SD* = 8.60) over familiar test trials (*M* = 10.38 s, *SD* = 7.23), *M*Δ = 5.06, [95% CI: 1.94, 8.19], *t*(19) = 3.39, *p* = .0030. There was no correlation between infants’ age and the magnitude of their preference; instead infants throughout the 3- to 4-month age range exhibited reliable novelty preferences. This striking reversal in test preferences from Experiment 2 was confirmed in a 2 (Experiment: 2 vs 4) x 2 (Test Trial: Familiar versus Novel) ANOVA which identified a significant cross-over interaction between Experiment and Test Trial, *F*(1,58) = 17.94, *p* < .0001.

We once again performed several complementary analyses to assess the robustness of infants’ rule learning from shapes. First, a Wilcoxon (non-parametric) signed rank test comparing infants’ looking between familiar and test trials found this to corroborate the Student’s *t*, V = 29, *p* = .0032. Second, infants dishabituated to novel test trials (*t*(19) = 3.23, *p* = .0044) but not to familiar trials (*t*(19) = .86, *p* = .40). Finally, linear mixed-effects models examining the influence of Test Type, Age, and Auditory Context on looking times revealed reliable main effects of Test Type (*β* = 4.69, *SE* = 2.05, *χ*^2^(1) = 5.11, *p* = .024) but no other effects or interactions. Infants had an overall reliable preference for novel trials; unlike Experiment 2, this preference did not change by age.

### Discussion

Infants’ success converges well with their success when presented with images of dogs (Experiment 2). In addition, the direction of their test preferences in Experiments 2 and 4 indicate that 3- and 4-month-olds successfully abstract rules in the visual modality, and that like adults, they encounter a greater processing load when the images are perceptually complex than when they are simple.

## General discussion

These experiments advance our understanding of the developmental origins of abstract rule learning. We have documented that both adults and infants can learn abstract rules in the visual modality, and that they do so best when the sequences of elements are spatially-structured and simultaneously-observable. Moreover, we find developmental continuity not only in the presentation format that best supports visual rule learning, but also in the processing load imposed by presenting sequences of complex (Experiments 1 and 2: images of dogs) and simple (Experiments 3 and 4: images of dogs) elements.

These findings challenge two claims about early rule learning. First, they constitute strong evidence against the hypothesis that rule learning is initially restricted to the auditory modality. Instead, from the first months of life, infants successfully learn abstract rules in both the visual and auditory modalities. Second, the current results reveal that stimulus familiarity alone cannot explain when infants succeed and fail to learn rules [14,17]. For both infants and adults, the complexity of the elements governs the processing burden–and, in turn, infants’ likelihood of success–in visual abstract rule learning.

Still, the current results leave one important question unresolved: *Why* do spatially-structured, simultaneously-observable sequences best support rule learning. One advantage of simultaneous observation is that it reduces demands on visual working memory [27]. When sequences are presented simultaneously, learners do not need to maintain in memory the visual features of earlier entities in order to establish their relation to later elements in a sequence. A second likely advantage is that simultaneous observation promotes comparison of first- and second-order relations amongst elements [28]. Finally, simultaneous observation provides an opportunity for Gestalt processes to organize the lower-level perceptual input into higher-level units [29–32]. Infants may organize sequences into two (for A-BB) or three (for A-B-A) groups that, in turn, support them in detecting the rule by aligning grouped elements for comparison [33].

Broadly speaking, we underscore the importance of considering the distinct properties of the distinct perceptual systems (e.g., auditory, visual) that interface between cognitive processes and the stimuli they take as input [34–35]. Humans are equipped with a host of domain-general learning mechanisms that, in principle, can operate over a wide variety of stimuli. But these mechanisms are not stimulus-neutral: stimuli that are most efficiently processed by the lower-level perceptual systems will most effectively engage those higher-level learning mechanisms.

## Supporting information

S1 Data Raw experimental data. Data from Experiments 1–4 is available for download. For infant experiments, by-trial and aggregated datasets are included.
(ZIP)
